# Physiologically Aggregated LacZ Applied in Trehalose Galactosylation in a Recycled Batch Mode

**DOI:** 10.3390/life13081619

**Published:** 2023-07-25

**Authors:** Martina Belkova, Tatiana Janegova, Eva Hrabarova, Jozef Nahalka

**Affiliations:** 1 Institute of Chemistry, Centre for Glycomics, Slovak Academy of Sciences, Dubravska Cesta 9, SK-84538 Bratislava, Slovakia; chemmbel@savba.sk (M.B.);; 2 Institute of Chemistry, Centre of Excellence for White-Green Biotechnology, Slovak Academy of Sciences, Trieda Andreja Hlinku 2, SK-94976 Nitra, Slovakia

**Keywords:** β-galactosidase, galactooligosaccharides, LacZ, in vivo enzyme immobilization, active inclusion bodies, alginate beads, encapsulation, magnetization, trehalose galactosylation

## Abstract

Galactooligosaccharides obtained via β-galactosidase transgalactosylation have health-promoting properties and are widely recognized as effective prebiotics. Trehalose-based galactooligosaccharides could be introduced into food and pharmaceutical industries similarly to trehalose. In light of this, new technological approaches are needed. Recently, in vivo enzyme immobilizations for recombinant proteins have been introduced, and physiological aggregation into active inclusion bodies (aIBs) has emerged as one such method of in vivo immobilization. To prepare LacZ β-galactosidase in the form of aIBs, we used a short 10 amino acid aggregation-prone tag. These native protein particles were simply washed from the cell lysate and applied in trehalose galactosylation in a recycled batch mode. In this study, aIBs entrapped in alginate beads, encapsulated in alginate/cellulose sulfate/poly(methylene-co-guanidine) capsules and magnetized were compared with free aIBs. Alginate/cellulose sulfate/PMCG capsules showed more suitable properties and applicability for biotransformation of trehalose at its high concentration (25%, *w*/*v*) and elevated temperature (50 °C).

## 1. Introduction

In *Escherichia coli*, LacZ gene codes β-galactosidase enzyme (E.C. 3.2.1.23) which hydrolyses terminal non-reducing β-D-galactose residues in β-D-galactosides. This enzyme (LacZ) is one of the most thoroughly characterized enzymes: the protein played a central role in Jacob and Monod’s development of the operon model for the regulation of gene expression [1] and belongs to a significant food industrial enzyme family utilized in the production of lactose-free dairy products [2]. *E. coli* is often preferred as an indicator organism to assess fecal contamination of food and water, however, LacZ activity can reflect the concentration of cells and β-galactosidase is often used generally as a marker for coliforms [3]. In humans, intestinal lactase (β-galactosidase) appears to be constitutive and yet ~70% of adults are deficient in this enzyme, which is required for the digestion of lactose [4]. Interestingly, β-galactosidase secreted by *Streptococcus thermophilus* in the intestine can inhibit colorectal tumorigenesis [5].

LacZ and other β-galactosidases demonstrate transgalactosylation activity, wherein the galactose from lactose is transferred to an acceptor other than water (such as lactose, glucose, galactose or trehalose) and di-, tri-, tetra- and higher galactooligosaccharides (GOS) are formed. GOS based on lactose have health-promoting properties and are widely recognized as effective prebiotics that lack toxicological effects and have been declared safe in EU and US jurisdictions [6]. Experimental studies have shown that the tri- and tetrasaccharide components of GOS are resistant to digestion, while disaccharides can be partially digested, and that β(1–6)-linked galactose in GOS is much more stable than β(1–4)-linked [6]. Isolation of GOS from natural sources is not cost-effective due to their relatively low concentration, and chemical synthesis (acid hydrolysis of lactose at elevated temperatures) is not preferred due to the possible formation of undesirable by-products [7]. Commercial GOS products are usually manufactured in batch fashion, and as such, the transgalactosylation is initiated in a stirred tank reactor with the addition of (semi-)purified soluble β-galactosidase at optimal reaction conditions (pH, temperature and substrate concentration) [7]. To improve the efficiency of commercial GOS production, various immobilized β-galactosidases have been used in sequential batch operations in stirred tank reactors, in continuous operations in packed reactors, and to a lesser extent, in continuously operated stirred tank reactors [8]. For example, *Aspergillus oryzae* β-galactosidase immobilized in glyoxyl agarose retained 75% of its initial activity at the end of the tenth batch [9], *Bacillus circulans* β-galactosidase immobilized in Eupergit C250L retained 60% of its initial activity after 15 sequential batches [10], and *A. oryzae* β-galactosidase immobilized on cotton cloth in a plug flow reactor continually operated for 48 days at 50 °C [11]. So far, a wide range of methods and carriers for immobilizing β-galactosidase or whole cells have been studied and compared, have been developed [12,13,14]. However, much more β-Galactosidase has been produced via recombinant technology in recent years [15]. In the case of the recombinant enzyme production, the fusion of aggregation-prone tags has emerged as a solution for in vivo enzyme immobilization in industrial biocatalysis, as a method of physiological aggregation of the enzyme directly inside the host cell [16,17,18,19]. In this study, a very short (10 amino acid) aggregation-prone tag was fused to LacZ, and the resulting protein was overexpressed in *E. coli* host cells as active inclusion bodies (aIBs). aIBs can be directly recovered from the reaction mixture via centrifugation, however, handling the micro- to nano-sized gelatin aIBs is difficult on a large industrial scale. aIBs can be better separated from the reaction mixture when a method of whole cell immobilization is additionally applied to them. Thus, in this study, we compared entrapment, encapsulation, glutaraldehyde crosslinking, and magnetization of LacZ aIBs (LacZ-IBs) [20].

IBs, or physiologically aggregated peptides/proteins, which arise from mistranslation or other errors in translational and post-translational processes, are generally considered to be harmful in the elderly, and are mainly involved in neurodegenerative diseases [21]. For example, aggregation of β-amyloid peptide is mainly responsible for Alzheimer’s disease, while aggregation of α-synuclein is responsible for Parkinson’s disease and other synucleinopathies [21,22,23]. Oral administration of the diglucose derivative trehalose (Treh) reduces neurodegeneration and improves motor and cognitive performance in mouse models, albeit at a high concentration [24,25]. Treh is not resistant to digestion in the human body, as it is broken down in the small intestine by trehalase, a brush border enzyme that hydrolyzes the α,α-1,1-glycosidic bond in trehalose into two glucose molecules. Treh at concentrations of up to 50 g in 400 mL of water per day has been shown to be safe for human consumption and has been declared safe in EU and US jurisdictions [26]. In light of this, Treh is widely used as a food stabilizer; it has been incorporated into more than 8000 foods, mainly air-dried and frozen foods [26]. However, Treh may have various effects on the gut microbiome and appears to play a role in the pathogenicity of some microbes. For example, it has been speculated that consumption of Treh may be a risk factor for epidemic strains of *Clostridioides difficile* [27]. Thus, research on Treh analogs that are resistant to degradation by gut microbiome trehalases or intestinal trehalase could be beneficial for their use in combination with Treh in the food industry and in medicine [28]. *Escherichia coli*, *Bacillus circulans* and *Aspergillus oryzae* β-galactosidases were used for the conversion of Treh into GOS [29,30]. Trisaccharides (TrehGOS) were the most abundant oligosaccharides, and NMR analyzes revealed that galactose monomers are linked by β-(1→6) and β-(1→4) linkages to trehalose [29,30]. LacZ produced β-(1→6) and β-(1→4) TrehGOS variants in a ratio of 9:1 [29]. In this study, we focused on the production of β-(1→6) TrehGOS by LacZ-IBs entrapped/encapsulated in a polysaccharide matrix.

## 2. Materials and Methods

### 2.1. Materials

High viscosity sodium alginate (Alg) with a 60% D-mannuronic acid content was obtained from ISP Alginates (Girvan, Ayrshire, UK). Cellulose sulfate (CS, sodium salt) was obtained from Acros Organics (Geel, Belgium). Poly(methylene-co-guanidine), hydrochloride (PMCG) was obtained from Scientific Polymer Products, Inc. (Ontario, NY, USA) as a 35% aqueous solution and was lyophilized after delivery.

### 2.2. Cloning, Expression and Isolation of LacZ-IBs

Codon optimization for *E. coli*, gene synthesis, and cloning (NdeI/BamHI) with the pET-30b (+) plasmid were performed using GenScript. The LHSAKIVVIG tag [31] was inserted between the initial methionine and threonine in the LacZ amino acid sequence (https://www.genome.jp/entry/eco:b0344; accessed on 21 July 2023). His-tag was inserted at *C*-terminus linked by two serines to the end lysine. Chemically competent *E. coli* BL21 (DE3)T1R cells were transformed with the plasmid and cultivated on kanamycin/LB/agar plates. The colonies were regrown in 30 mL LB medium supplemented with kanamycin (30 μg/mL) for approximately 20 h at 32 °C; then, 15 mL of the solution was transferred to a 300-mL flask with 100 mL LB-media and incubated for 4 h at 37 °C with shaking at 150 rpm. Recombinant expression was induced by adding an inductor (isopropyl β-D-1-thiogalactopyranoside; 400 μM) for 20 h at 15–20 °C, at 150 rpm. After centrifugation (45 min, 9700× *g*, 4 °C), the biomass was suspended in a minimum volume of water, frozen, and lyophilized. The lyophilized cells were stored at −25 °C until further use. Lyophilized cells were lysed twice in BCL (CelLytic B Cell Lysis Reagent; Sigma-Aldrich, St. Louis, MO, USA) in a ratio of 1 mg of cells/50 μL of BCL solution. After centrifugation of the lysate (5 min, 21,000× *g*, 4 °C), cell debris was washed three times via centrifugation using the same ratio of Tris-HCl buffer (50 mM, pH 7.5). After the final centrifugation step, IBs were resuspended in the same volume of Tris-HCl buffer and stored at 6 °C. The purity and quality of LacZ-IBs was measured by SDS-PAGE, and the intensity of bands was evaluated by UVP Doc-It LS Analysis Software, version 1 (Thermo Fisher Scientific, Waltham, MA, USA).

### 2.3. Entrapment of LacZ-IBs and Whole Cells into Alg Beads

Sodium alginate (2.5% *w*/*v*, 60% D-mannuronic acid) was mixed with aIBs (isolated from 5 mg of lyophilized cells) or with lyophilized cells (10 mg) to a final volume of 2 mL in deionized water and left at 4 °C overnight. Next, the mixture (1 mL) was dropped through a nozzle into a stirred gelation bath with 1% CaCl_2_ × 2H_2_O solution (25 volumes of alginate solution). A coaxial air stream was used to blow the droplets from the needle tip into the gelling bath. After 45 min, the alginate beads were decanted and mixed with 4.0 mL of the reaction solution (25% trehalose, 10% lactose, 10 mM MgCl_2_, 50 mM Tris-HCl, pH 7.5) and stored in a refrigerator. In the case of glutaraldehyde crosslinking, alginate beads were exposed for 5 min in ten volumes of 0.5% glutaraldehyde solution and washed three times in 50 mL of 50 mM Tris-HCl, pH 7.5 [32].

### 2.4. Encapsulation of LacZ-IBs and Whole Cells into Alg/CS/PMCG Capsules

The polyanion solution contained 1% (*w*/*v*) each of alginate and cellulose sulfate, 0.9% (*w*/*v*) NaCl, LacZ-IBs from 2.5 mg of lyophilized cells or whole cells (10 mg) and was mixed and finalized at the resulting volume of 2 mL in water and left overnight at 4 °C. The mixture was subsequently dropped through a nozzle using a coaxial air stream into 10 volumes of a stirred polycation solution consisting of 2% (*w*/*v*) PMCG, 1.0% (*w*/*v*) CaCl_2_ × 2H_2_O and 0.9% (*w*/*v*) NaCl in water at room temperature. Immediately after 30 to 60 s of incubation, the reaction was stopped via 20-fold dilution with water. The capsules were decanted and mixed with 4.0 mL of the reaction solution (25% trehalose, 10% lactose, 10 mM MgCl_2_, 50 mM Tris-HCl, pH 7.5) and stored in a refrigerator.

### 2.5. Magnetic Modification of LacZ-IBs

Magnetic particles were prepared by mixing of FeSO_4_ × 7H_2_O and NiSO_4_ × nH_2_O in a ratio of 9:1 g/L in 100 mL of deionized H_2_O. pH was adjusted to 12 by a dropwise addition of 1 M KOH and after the formation of a dark metal precipitate, the solution was diluted in 1:1 with dH_2_O and exposed to microwaves (960 W) for 10 min. After cooling down, formed particles were rewashed several times using dH_2_O until reaching neutral pH. 500 µL of particles were washed three times via Tris-HCl buffer (50 mM, pH 7.5) and mixed with 2.5 mg of LacZ-IBs from cells in 1 mL of the buffer. The mixture was then frozen at −25 °C for one week and then lyophilized (0.1 mBar, 45 min, −10 °C, without final drying). Modified LacZ-IBs were mixed with 4.0 mL of the reaction solution (25% trehalose, 10% lactose, 10 mM MgCl_2_, 50 mM Tris-HCl, pH 7.5) and stored in a refrigerator.

### 2.6. Transgalactosylation by LacZ-IBs and TLC-Monitoring

**Figure 1:**
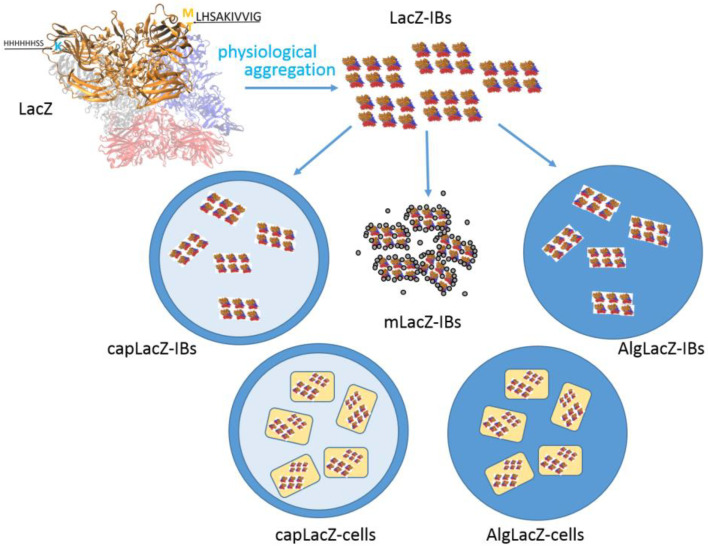
Scheme representing the experimental design.

**Figure 2:**
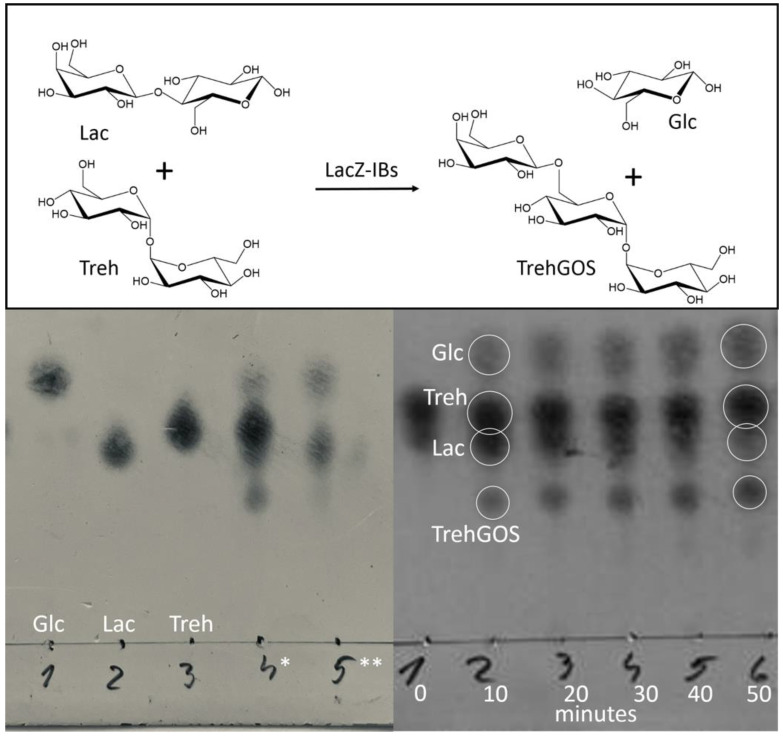
Transgalactosylation and TLC-monitoring. Lac—lactose (β-D-galactopyranosyl-(1→4)-D-glucose); Glc—glucose; Treh—trehalose (α-D-glucopyranosyl-(1→1)-α-D-glucopyranoside); and TrehGOS—the main (90%) product (β-D-galactopyranosyl-(1→6) α-D-glucopyranosyl-(1→1)-α-D-glucopyranoside). * Reaction. ** Reaction without trehalose.

The nomenclature and design of the biocatalysts are summarized in Table 1 and Figure 1. The reaction (Figure 2) was carried out at 50 °C at 130 rpm in 5 mL of the mixture in 50 mL Corning centrifuge tubes. Samples were diluted 1:50 and spotted onto a silica gel plate. Each sample was applied at least three times. The plates were then placed in beakers with a mobile phase composed of n-butanol, acetic acid and water in a ratio of 2:1:1. The plates were briefly dried with a heat gun and Thymol solution (Thymol, sulfuric acid, ethanol; 0.5 g, 5 mL, 95 mL) was subsequently applied to visualize the spots while the plates were dried again. The intensity of spots was evaluated via UVP Doc-It LS Analysis Software, version 1 (Thermo Fisher Scientific, Waltham, MA, USA) (Figure 2).

**Table 1:** Nomenclature of biocatalysts.

LacZ	β-galactosidase enzyme
LacZ-IBs	active inclusion bodies of LacZ
free LacZ-IBs	LacZ-IBs recovered from the reaction mixture by centrifugation
AlgLacZ-IBs	LacZ-IBs entrapped into calcium alginate beads (Alg)
capLacZ-IBs	LacZ-IBs encapsulated into Alg/CS/PMCG capsules
mLacZ-IBs	magnetized LacZ-IBs
AlgLacZ-cells	whole cells expressing LacZ-IBs entrapped into Alg-beads
capLacZ-cells	whole cells expressing LacZ-IBs encapsulated into Alg/CS/PMCG-capsules

## 3. Results and Discussion

**Figure 3:**
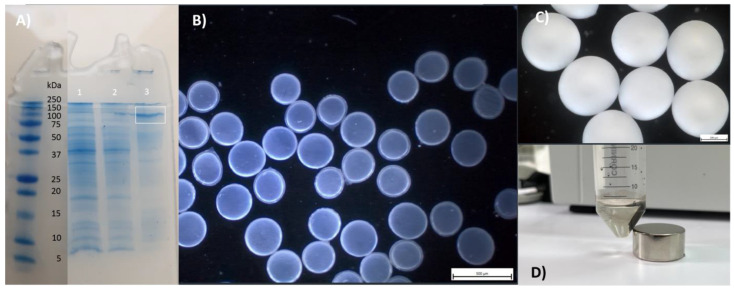
Immobilization of *E. coli* LacZ (116 kDa). (**A**) SDS PAGE, 1 mg cells/100 µL lysate, lane 1—supernatant of the cell lysate (2× diluted); lane 2—supernatant of the cell lysate (5× diluted); lane 3—sediment/aIBs (10× diluted). (**B**) Alg/CS/PMCG capsules after 25 rounds, 500 µm size indicator. (**C**) Alg-beads after 25 rounds, 200 µm size indicator. (**D**) Magnetized aIBs.

Sodium dodecyl sulfate-poly acrylamide gel electrophoresis was used to compare soluble and insoluble fractions of cell lysates. Figure 3a shows the pull-down result of LacZ. As demonstrated in the figure, LacZ was expressed as IBs using LHS1, 0.4 mM IPTG (isopropyl β-d-1-thiogalactopyranoside) for 20 h at 15–20 °C. LacZ monomer has 116 kDa [33]. To obtain active LacZ inclusion bodies, we used the short tag LHS1. This ten-amino acid aggregation-prone tag was designed in our previous work, where a combination of short sequences from the *Escherichia coli* polyphosphatase ygiF was used for pull-down into aIBs [31]. As was shown previously, the minimal LHS1-tag was sufficient for 90–100% pull down into aIBs when the untagged recombinant protein is not highly soluble and partially enters IBs during overexpression, while a 32-amino acid LHS2-tag was needed for highly soluble tuGFP [31]. LacZ is a soluble protein when a standard 0.4 mM IPTG inductor and temperature of 20 °C are used. However, when IPTG is increased to 1 mM and the temperature is maintained at 37 °C for 20 h, aIBs of LacZ can be obtained [34]. In our laboratory experiment, however, we found that protein/peptide “pull-down” fusion and slow expression below 20 °C provide the best activities of aIBs, and therefore we used an LHS1-tag, 0.4 mM IPTG for 20 h at 15–20 °C. In the case of LacZ, artificial inclusion bodies (artIBs) were also studied [35]. ArtIBs are comprised of pure proteins, and their fabrication is based on the coordination of divalent cations (400 mM) to proteins containing histidine tags. In the case of LacZ, the Zn^2+^ ion untraditionally caused the deactivation of the enzyme. However, a mixture of Ca^2+^ (an aggregator) and Mg^2+^ (an enzyme activator) in a ratio of 2:1 offered the best performance for artIBs catalytic particles [35]. Theoretically, artIBs could provide the best activities, but they must be prepared from isolated/purified enzymes and the toxicity of divalent cations usually increases with temperature. Due to better sugar solubility, transgalactosylation by β-galactosidases takes place at 50 °C and at high donor/acceptor concentrations. Nevertheless, the artIBs experiment showed that Ca^2+^ is an excellent LacZ linker and its inactivating effect is only moderate compared to that of Zn^2+^ [35]. In light of this, entrapping/encapsulating in Alg-gel beads, where 100 mM Ca^2+^ is typically used for gelation, could provide an excellent matrix for LacZ-IBs. We prepared aIBs of LacZ and studied their entrapment/encapsulation in alginate gel beads/capsules. We subsequently used three protocols for the additional immobilization of aIBs: entrapment to Alg-beads with or without additional glutaraldehyde crosslinking; encapsulation into Alg/CS/PMCG capsules; and magnetization [20]. These three immobilizations were compared with free LacZ-IBs and entrapped/encapsulated whole cells.

25 to 30% (*w*/*v*) Treh and 15 to 25% (*w*/*v*) lactose are used in the transgalactosylation required for TrehGOS production [29,30]. In our experiments, lactose was reduced to 10% to prevent the production of lactose based GOS, and the relatively expensive Treh was maximized at 25%. Reactions were performed in 5 mL at 50 °C and a pH level of 7.5. 10 mM of MgCl_2_ was present as an enzyme activator. At these conditions, LacZ-IBs had excellent specific activity (309 µmol/min/mg_LacZ-IBs_). Since *E. coli* reaches a density of 6 g dry cell weight per liter of culture medium during growth in the bioreactor and LacZ-IBs were overexpressed at 0.255 ± 57 mg/mg dry cells, up to 472,770 U of LacZ can be obtained from this liter culture. Despite different substrates, this volumetric activity is far from recombinant: β-galactosidase from *Kluyveromyces lactis* and *Aspergillus niger* was produced in *Saccharomyces cerevisiae* at values of 17,000–40,000 U/mL and 2754–7350 U/mL, respectively [15]. However, for example, *Kluyveromyces* sp. β-galactosidase produced in *E. coli* (pET-30a (+)) reaches 40,000 U/L (in shake flasks) [36].

Alg-beads were originally proposed as an entrapment matrix for whole cells, due to the fact that the open lattice structure leads to the leakage of large molecules such as proteins, and the porosity of Ca^2+^ Alg-gel ranges from 5 nm to 200 nm in diameter [37]. Entrapment into Alg-beads with a combination of glutaraldehyde crosslinking was proposed as an additional method for more effective manipulation of aIBs [32]. Currently, there are various Alg-dropping-technologies for scale-ups, and this immobilization technique is established in the industry. For example, Alg-beads can be mass-produced via a jet cutting method [38,39]. In light of this, we entrapped LacZ-IBs in the Alg-beads (Figure 3c). Entrapped LacZ-IBs (AlgLacZ-IBs) showed an apparent specific activity of 92 µmol/min/mg_LacZ-IBs_, however, up to 90% of the activity was lost after glutaraldehyde crosslinking, and therefore only glutaraldehyde-free beads were used in further experiments. Alg/CS/PMCG capsules were originally developed for encapsulation and transplantation of pancreatic islets [40], but can also be used for the encapsulation of aIBs used in biotransformations [41]. A technique for larger-scale production exists; a special chemical reactor has been designed for the continuous production of uniform Alg/CS/PMCG capsules [42]. In light of this, we encapsulated LacZ-IBs in Alg/CS/PMCG capsules (capLacZ-IBs, Figure 3b). Encapsulated LacZ-IBs showed an apparent specific activity of 216 µmol/min/mg_LacZ-IBs_. Hydrophobic aIBs can also be magnetized very simply via adsorption of magnetic iron oxide particles prepared via microwave synthesis from ferrous sulfate [43]. Magnetic iron oxide particles can be mass-produced by a continuous flow microwave reactor [44], and in light of this we prepared iron/nickel (9:1) particles and magnetized LacZ-IBs (mLacZ-IBs) by them. mLacZ-IBs had an apparent specific activity of 75 µmol/min/mg_LacZ-IBs_. For comparison, we also entrapped and encapsulated whole cells (AlgLacZ-cells; capLacZ-cells), the apparent specific activities were 37 U/mg_LacZ-IBs_ and 21 U/mg_LacZ-IBs_, respectively.

**Figure 4:**
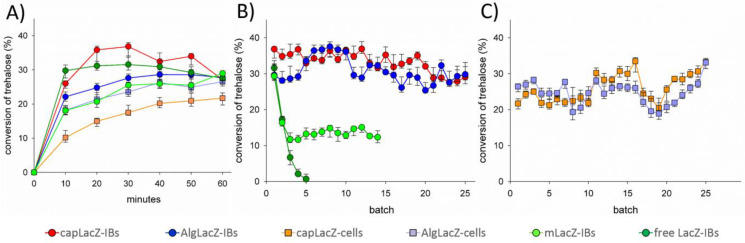
Trehalose galactosylation in a recycled batch mode. (**A**) Time course of the first cycle. (**B**) Recycled batches for free LacZ-IBs and entrapped/encapsulated/magnetized aIBs (AlgLacZ-IBs/capLacZ-IBs/mLacZ-IBs). (**C**) Recycled batches for entrapped/encapsulated cells (AlgLacZ-cells/capLacZ-cells).

Figure 4a shows the time courses of free and magnetized LacZ-IBs, entrapped/encapsulated aIBs, and entrapped/encapsulated whole cells. Free LacZ-IBs reached maximum transgalactosylation at 20–30 min, capLacZ-IBs at 30 min, AlgLacZ-IBs at 40 min, and mLacZ-IBs, AlgLacZ-cells, and capLacZ-cells at 60 min. Rapid transformations minimized lactose hydrolysis and allowed Treh conversion to reach 37% for capLacZ-IBs and 32% for free LacZ-IBs (Table 2).

Kim et al. [29] achieved 30% Treh conversion using soluble LacZ (800 IU in 100 mL), however, they used 30% (*w*/*v*) Treh and 15% (*w*/*v*) lactose at 45 °C for 48 h. To compare operational stability of the biocatalysts in the repetitive batches, one cycle for biotransformation was set up to 30 min for free and capLacZ-IBs, 40 min for AlgLacZ-IBs, and 1 h for mLacZ-IBs, AlgLacZ-cells, and capLacZ-cells. Entrapped/encapsulated LacZ biocatalysts were easily decanted from the reaction mixture (the last volume was pipetted off), and mLacZ-IBs were easily separated with a magnet (Figure 3d) and supplied with fresh reaction mixture. However, free LacZ-IBs had to be centrifuged (5 min, 21,000× *g*, 4 °C) and resuspended via pipette in fresh reaction mixture. Surprisingly, free LacZ-IBs completely lost activity after five cycles (Figure 4b). Absorbance at 280 nm and decreased sediment volume from centrifugation confirmed that LacZ-IBs were partially solubilized at 25% (*w*/*v*) Treh. Unlike other disaccharides, Treh sequesters water more effectively (according to the water exclusion theory), and as a result of this competition, water molecules are removed from the protein. Due to the positive free energy difference between the unfolded and native state, proteins are kept in their native conformation state [26]. In the case of mLacZ-IBs, this “refolding” process appeared to work only partially; the activity dropped to a constant value after the third cycle and the particles then maintained activity for another 12 cycles (Figure 4b). Encapsulation of LacZ-IBs in Alg/CS/PMCG capsules (capLacZ-IBs, Figure 3b) appears to provide the most effective solution: the Alg/CS/PMCG membrane is mechanically resistant, thin and highly permeable, and importantly, has a molecular weight cut-off of 116 kDa [45], which is exactly the size of the LacZ monomer [29]. The shift to native conformations and solubilization of aIBs by Treh occurs inside the capsules, and subsequently the LacZ tetramer remains inside the capsules. As shown, the capsules were not ruptured after 25 biotransformations (Figure 3b). Simple entrapment of LacZ-IBs into Alg beads also performed satisfactorily for 25 cycles, however, disaggregation of the protein by Treh actually increased the yield of TrehGOS in the first 10 cycles, at which point it is likely that enzyme leakage slowly reduced catalyst activity. In addition, swelling of the AlgLacZ-IBs particles was observed in the last cycles; it appears that 5–10 mM of Ca^2+^ should be added to the reaction mixture to improve the operational stability of the Alg-beads.

Although *E. coli* is considered the microorganism of first choice for the production of recombinant proteins, the toxic factors associated with coliform host cells have made “whole recombinant cells” unsuitable for use in the food industry, despite the fact that most of the strains used are harmless [15]. Nevertheless, we entrapped/encapsulated whole cells expressing recombinant LacZ-IBs. As shown in Figure 4c, TrehGOS yields varied much more between runs than for biotransformations by aIBs. Heat lysis/cell permeabilization (50 °C) appeared to increase initial activity and yields, and regrowth of cells inside the capsule/bead during storage (overnight and weekend in the refrigerator) reduced the performance of AlgLacZ-cells and capLacZ-cells (Figure 4c).

## 4. Conclusions

In this study, the physiological aggregation of LacZ applied in the galactosylation of trehalose was investigated. Insertion of a short 10-amino acid aggregation-prone sequence (LHS1-tag) between the initial methionine and threonine in the LacZ amino acid sequence resulted in a “pull-down” of enzyme expression (0.4 mM IPTG, 20 h, 15–20 °C) to super-active inclusion bodies (309 µmol/min/mg_LacZ-IBs_). However, these aIBs rapidly lost their volume and activity due to disaggregation via a reaction mixture with a high concentration of Treh (25%). Encapsulation of aIBs into Alg/CS/PMCG capsules (membrane cutoff 116 kDa) solved this problem, and the encapsulated aIBs can be used at least 25 times for the rapid galactosylation of Treh in a recycled batch mode. Crosslinked inclusion bodies (CLIB process [32]) did not work as expected; LacZ-IBs were sensitive to exposure for 5 min in 0.5% glutaraldehyde. Simple entrapment of aIBs into alginate beads is effective, however, leakage of LacZ and swelling of Alg-beads is observed. Magnetization via adsorption on iron/nickel oxide magnetic particles does not work well for LacZ-IBs, since the magnetic particles store insufficient amounts of aggregated enzyme, and transgalactosylation is too slow.

This study proves that LacZ can be pulled-down into active inclusion bodies via the short LHS1-tag inserted between initial methionine and threonine, and these aIBs can be effectively applied for the biotransformation of Treh into TrehGOS at an exact temperature (50 °C), a pH level of 7.5, and specific concentrations of substrates. However, this study did not compare the presented immobilization strategies in terms of the overall stability of the configurations in different pH levels, temperature ranges or other criteria. As a basic criterion for the selection of one preparation, a comparison of operational stability and performance in repeated batches was used in this experiment. In light of this, encapsulation into Alg/CS/PMCG-capsules (membrane cutoff value: 116 kDa) represents a fundamental choice. Simple entrapment into alginate beads may be applicable for biotransformation, and as mentioned in the introduction, enzyme leakage is not necessarily a problem, as β-galactosidase in the intestine has been shown to inhibit colorectal tumorigenesis [5], but in this case the reaction must be stopped via rapid cooling and calcium should be added to the reaction mixture to improve the mechanical stability of the Alg-beads. On the other hand, the leakage of the enzyme from the magnetic particles significantly slows down the reaction and the renders its performance too inefficient.

In summary, the LHS1-tag allows physiological aggregation of LacZ to obtain active inclusion bodies, which are simply washed from the cell lysate. Trehalose promotes the disaggregation of aIBs, but their encapsulation into Alg/CS/PMCG-capsules or entrapment into Alg-beads allows for their application in the galactosylation of trehalose in a recycled batch mode. Reactions were performed on a 5 mL scale, and therefore the next steps to follow in this research/process line will include testing the operational stability and performance of this experiment on a larger scale (5–50 L).

## Figures and Tables

**Table 2:** Maximal trehalose conversion and LacZ-IBs/Treh ratio used in each type of biotransformation.

Biocatalyst	Cells ^1^ (mg)	LacZ-IBs ^1^ (mg)	Max Treh Conversion (%)
free LacZ-IBs	1.00	0.255	32
AlgLacZ-IBs	2.50	0.638	29
capLacZ-IBs	1.25	0.319	37
mLacZ-IBs	2.50	0.638	26
AlgLacZ-cells	5.00	1.275	25
capLacZ-cells	5.00	1.275	21

^1^ The maximum amount of LacZ-IBs that could be immobilized was used. For example, due to hydrophobicity, only LacZ-IBs from 1.25 mg of cells could be encapsulated into 1 mL of Alg/CS/PMCG capsules compared to 5 mg of whole cells. 1 g of Treh in a volume of 4 mL and 1 mL of biocatalyst were used for biotransformation.

## Data Availability

Not applicable.
